# Trends in the Mortality, Deaths, and Aetiologies of Lower Respiratory Infections Among 204 Countries from 1991 to 2021: An Updated Systematic Study

**DOI:** 10.3390/v17070892

**Published:** 2025-06-25

**Authors:** Meichen Li, Min Liu, Jue Liu

**Affiliations:** 1 Department of Epidemiology and Biostatistics, School of Public Health, Peking University, Beijing 100191, China; 2411120005@stu.pku.edu.cn (M.L.); liumin@bjmu.edu.cn (M.L.); 2 Key Laboratory of Epidemiology of Major Diseases (Peking University), Ministry of Education, Beijing 100191, China; 3 Institute for Global Health and Development, Peking University, Beijing 100871, China

**Keywords:** lower respiratory infections, deaths, ASMR, aetiology

## Abstract

Lower respiratory infections (LRIs) persist as a major global health threat. This study analyses the 1991–2021 trends in LRI mortality, deaths, and aetiologies across 204 countries using Global Burden of Disease 2021 data, aiming to evaluate the disease burden of LRIs and provide evidence-based guidance for prevention strategies. To quantify the temporal trends, the annual percentage change was estimated (EAPC) using linear regression modeling. Globally, the ASMR for LRI decreased by an average of 2.29% annually (95% CI: 2.16–2.42%). While ASMR decreased in 20 of the GBD regions, mortality rates in Southern Latin America increased (EAPC = 1.32, 95% CI: 0.98–1.67). The LRI burden remains the heaviest in low SDI regions and sub-Saharan Africa. LRIs continue to cause high mortality in children and the elderly. Mortality in children decreased rapidly, while mortality in the elderly declined more slowly. *Streptococcus pneumoniae* was the leading cause of LRI-related deaths, followed by *Staphylococcus aureus* and *Klebsiella pneumoniae*. LRIs remain a leading cause of global mortality, especially in low SDI regions, and among children and the elderly. Future research on LRIs and the development of effective prevention and control strategies are essential to reduce the disease burden of LRIs.

## 1. Introduction

Lower respiratory infections (LRIs) are infections affecting the structures below the larynx, including the trachea, bronchi, bronchioles, and lungs (e.g., bronchitis, bronchiolitis, and pneumonia), and are one of the leading contributors to global disease burden and mortality [1,2]. According to the World Health Organization (WHO), LRIs rank among the top ten leading causes of death globally [2]. In children under five years of age, LRI is one of the most common causes of death [3,4], especially in low- and middle-income countries, where the mortality rate due to LRIs remains high [5,6]. Among the elderly and individuals with compromised immune systems, LRIs are prone to causing severe complications, significantly increasing the risk of mortality [7]. In 2019, pneumonia and other lower respiratory infections collectively constituted the most lethal category of communicable diseases. As the fourth leading cause of death globally, they accounted for at least 2.49 million fatalities. According to WHO reports, in 2021, lower respiratory infections were the leading cause of death in low-income countries. Therefore, it is crucial to investigate the global disease burden of LRIs and its distribution across different populations and regions, as well as formulate appropriate prevention and control strategies.

In recent years, epidemiological studies on LRIs were conducted in several countries, revealing insights into its aetiology, mortality, and disease burden [8,9,10]. Although these studies have provided important information regarding the aetiology, mortality, and disease burden of LRI, systematic analyses of the global aetiology, deaths, and mortality associated with LRI over the past 30 years remain limited. Many existing studies are restricted to specific regions or age groups [10], or utilize short-term cross-sectional data [9], failing to provide a long-term and comprehensive global disease burden analysis. Additionally, although studies have revealed the impact of the COVID-19 pandemic on the epidemiological patterns and mortality of respiratory diseases [11] there is currently no comprehensive report on the burden of LRIs in terms of aetiologies, deaths, and mortality globally since the pandemic. Our team has previously conducted research on the burden of LRIs from 1990 to 2019 [12], but this study did not incorporate data on the burden of LRIs during the COVID-19 pandemic and the impact of the COVID-19 pandemic on the global burden of LRIs has not been fully investigated. Therefore, it is essential to promptly assess the post-pandemic burden of LRIs, especially the changes in aetiology, mortality and deaths, which are crucial for the development of global LRI prevention and control strategies.

Our analysis encompassed 204 countries and territories categorized under the World Bank income classification system, with these regions further grouped into 21 geographic sub-regions based on the GBD framework. This stratified geographical approach enables the current study to provide an updated analysis of the trends in deaths, mortality, aetiology, and the disease burden of LRI among these 204 countries spanning from 1991 to 2021 in the post-COVID-19 pandemic era, elucidating mortality and the aetiological burdens while generating evidence for preventive measures.

## 2. Materials and Methods

### 2.1. Data Collection

We collected data on the number of LRI deaths and age-standardized mortality rates (/100,000) (ASMR) from 1991 to 2021 categorized by age group, sex, region, and country, from the GBD result tool (2021 version) [13]. Additionally, this study collected data on the number of deaths and ASMR for 18 high-burden aetiologies of LRI (compared with only 4 aetiologies included in previous studies) [12]. A standardized approach for estimating deaths and mortality was provided by the GBD study, detailed in previous studies [5]. The SDI of different regions, countries, and territories were extracted from the GBD 2021 Socio-Demographic Index (SDI) 1950–2021. SDI is a composite indicator of development status strongly correlated with health outcomes, which is the geometric mean of 0–1 indices of total fertility rate under the age of 25 (TFU25), mean education for those ages 15 and older (EDU15+), and lag-distributed income (LDI) per capita. All 204 countries and territories were classified into five regions according to the SDI levels, including low, low-middle, middle, high-middle, and high SDI regions. In the meantime, 204 countries and territories were also grouped into 21 GBD regions based on their epidemiological homogeneity and geographical proximity, including East Asia, Andean Latin America, and others in the GBD study. In the analysis of the age-specific distribution of disease burden of LRIs, we organized the data of incidence and death rates into several age groups encompassing <5 years, 5–14 years, 15–49 years, 50–69 years, and 70+ years.

### 2.2. Statistical Analysis

This study describes the annual number of deaths and ASMR with their 95% uncertainty intervals (UIs) due to LRIs from 1991 to 2021 across 204 countries and regions, which were categorized into 21 GBD regions and 5 different SDI (Socio-Demographic Index) regions (Table S1). The percentage change in the number of deaths and the estimated annual percentage changes (EAPC) in ASMR were calculated to assess the trends in LRI-related deaths and mortality rates. The change in the number of deaths was calculated using the formula: (Deaths_2021_ − Deaths_1991_)/Deaths_1991_ × 100%. The value of EAPC was obtained by fitting the linear regression line using y = α + βx + ε, where y represents ln(rate) and x refers to the calendar year [14]. The value of EAPC = 100 × (exp[β]−1) and its 95%CI is attainable in the regression model [15]. In addition, the differences in the 18 aetiologies of death related to LRIs across various age groups, regions, and genders were also compiled. All statistical analyses in this study were conducted using R4.1.1.

## 3. Results

### 3.1. Global and Regional Trends in the Number of Deaths Due to LRIs and Its Mortality from 1991 to 2021

**Figure 1:**
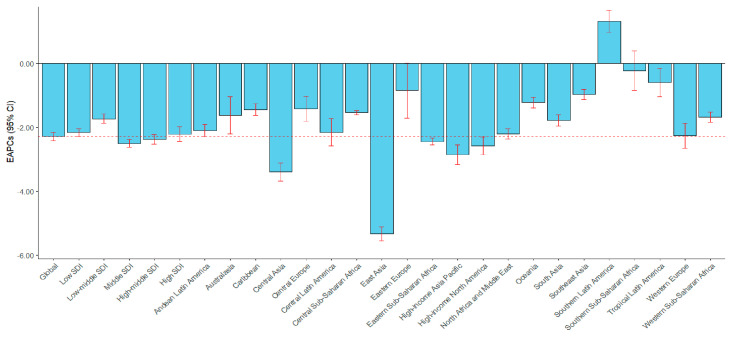
The EAPCs of lower respiratory infection (LRI) mortality from 1991 to 2021 by region. EAPC, estimated annual percentage change; SDI, Socio-Demographic Index.

At the global level, the number of deaths due to LRIs decreased from 2,976,802 (95% UI: 2,710,043–3,242,877) in 1991 to 2,183,001 (95% UI: 1,979,915 to 2,360,084) in 2021, a decrease of 26.67%. Regarding SDI regions, the low-middle SDI region had the highest number of deaths due to LRIs in 1991, with 940,472 people (95%UI: 844,161 to 1,040,618), and its ASMR was also the highest at 80.46 people per 100,000 (95%CI: 73.31 to 87.33). During 1991–2021, the number of deaths due to LRIs in low, low-middle, and middle SDI regions decreased by 34.31%, 36.83%, and 28.46%, respectively. However, in high-middle and high SDI regions, it increased by 0.21% and 11.47%, respectively. The mortality rates all showed a downward trend. Among them, the middle SDI region had the largest decrease, with EAPC = −2.50 (95% CI: −2.62 to −2.37, Table 1 and Figure 1).

For GBD regions, South Asia had 793,624 deaths (696,348 to 881,322) in 1991, which was the highest among GBD regions. In 1991, Central Sub-Saharan Africa had the highest LRIs mortality rate (168.81/100,000), and Eastern Europe had the lowest mortality rate (11.88/100,000). During 1991–2021, the region with the largest increase in the number of deaths due to LRIs was Southern Latin America (137.17%), and the region with the largest decrease was Central Asia (62.56%). The ASMR in Southern Latin America showed an upward trend with EAPC = 1.32, 95% CI: 0.98–1.67. The ASMR in all other GBD regions showed a downward trend (EAPCs = −5.33 to 0.22, Table 1 and Figure 1).

**Figure 2:**
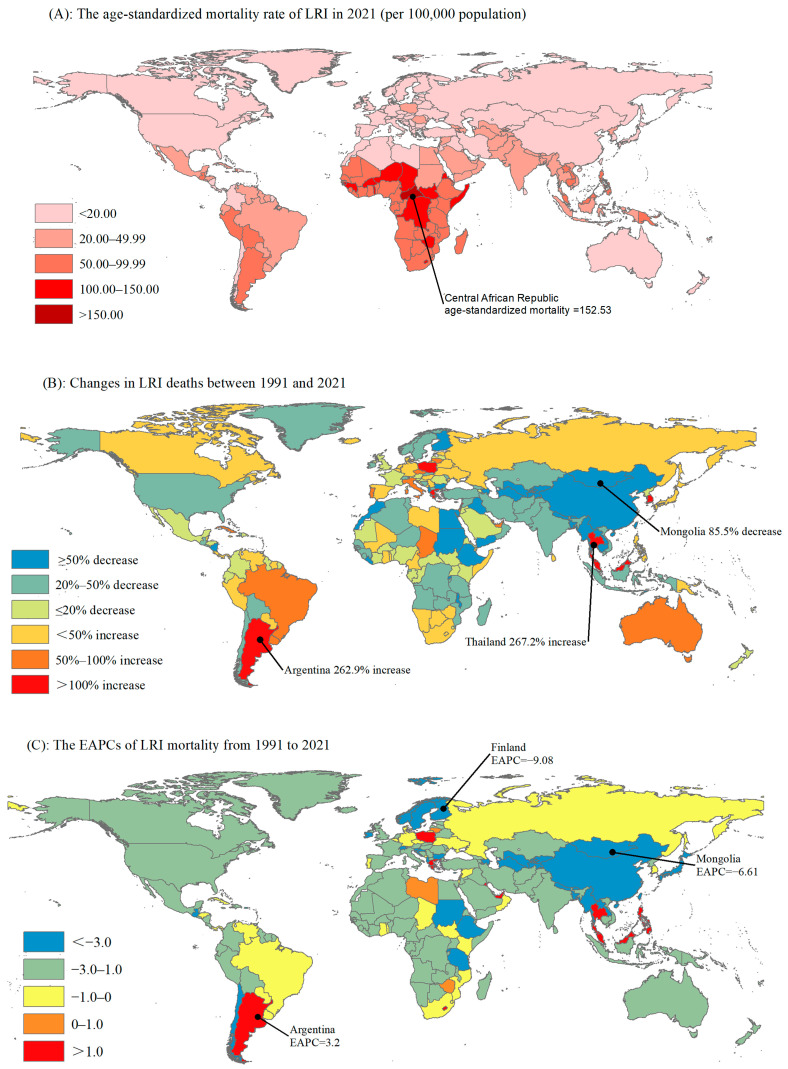
The ASMR, number of deaths, and EAPC of LRIs in 204 countries and regions. ASMR, age-standardized mortality rate; EAPC, estimated annual percentage change.

Globally, the ASMR decreased from 60.57 deaths per 100,000 people (95% CI: 55.82 to 65.10) in 1991 to 28.67 people (95% CI: 25.92 to 31.07) in 2021, with an average annual decrease of 2.29% (95% CI: −2.42 to −2.16) (Table 1). Among the 204 countries and territories in 2021, the ASMR for LRIs in the Central Asian republics is the highest, at 152.53 per 100,000 (Table S2 and Figure 2A). From 1991 to 2021, the number of deaths due to LRIs showed a declining trend in 122 countries, with the fastest decrease observed in Mongolia, which declined by 85.5%. Additionally, 82 countries exhibited an increasing trend, with the fastest increases seen in Kuwait and Thailand, which rose by 323.9% and 267.2%, respectively (Table S3 and Figure 2B). The two countries with the highest number of LRI-related deaths in 2021 were India (568,995) and China (446,448). Furthermore, we also calculated the EAPC values for each country. Finland had the fastest decline in age-standardized mortality, with an EAPC = −9.08 (95% CI: −10.34, −7.81), whereas Argentina showed a significant increasing trend, with an EAPC = 3.2 (95% CI: 2.71, 3.68, Table S4 and Figure 2C).

### 3.2. Trends in the Number of LRI Deaths and Mortality Rates Among Different Age Groups

**Figure 3:**
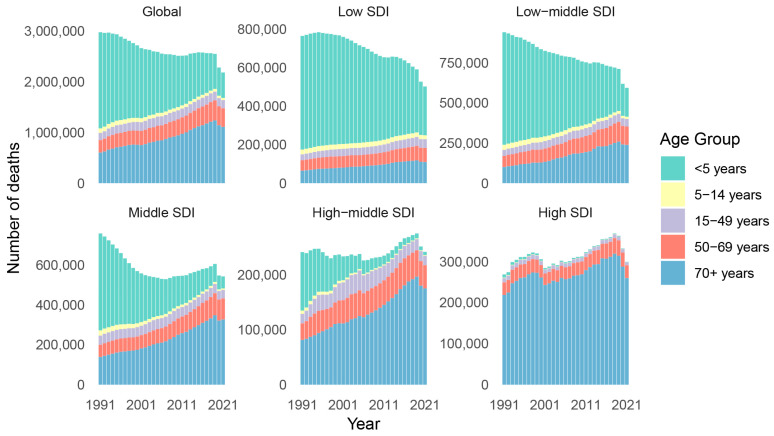
The deaths from LRIs by age group in global and SDI regions from 1991 to 2021. SDI, Socio-Demographic Index.

Significant disparities in the trends of the number of LRI deaths and mortality rates were observed among different age groups. The number of deaths due to LRIs in those under 5 years of age declined from 1,895,978 in 1991 to 501,909 in 2021, representing a decrease of 73.53%, followed by the number of deaths in the 5–14 years age group, which decreased from 88,755 to 43,764, a reduction of 50.69%. However, in other age groups, an upward trend was manifested. In low SDI and low-middle SDI regions, the variation trends in all age groups were in line with the global trend. In high-middle SDI and high SDI regions, the number of deaths due to LRIs in the population under 50 exhibited a downward trend, but the number of deaths caused by LRI in the middle-aged and elderly aged 50 and above presented an upward trend. In various regions, especially among the elderly over 70 years old, the upward trend in the number of deaths resulting from LRIs was severe, considering that this was highly likely to be related to COVID-19 infection (Table S5 and Figure 3).

**Figure 4:**
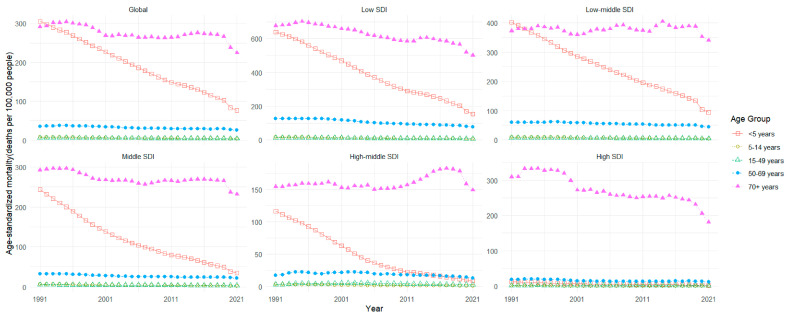
The mortality rates of LRIs by age group in global and SDI regions from 1991 to 2021. SDI, Socio-Demographic Index.

From 1991 to 2021, the global LRI mortality rate decreased from 60.5 to 28.67 per 100,000 (EAPC = −2.29, 95% CI: −2.42 to −2.16). The number of deaths among children under 5 years old decreased from 1,895,978 in 1991 to 501,909 in 2021, a decrease of 73.53%. The mortality rate decreased from 305.12 to 76.26 per 100,000 (EAPC = −4.19, 95% CI: −4.47 to −3.92), showing a downward trend, and the rate of decline was faster than that of the population. As can be seen from Figure 3, in low SDI and low-middle SDI regions, the LRI mortality rate among children under 5 years old is still very high. The number of deaths among the elderly aged 70 and above increased from 604,209 in 1991 to 1,110,706 in 2021, an increase of 83.83%. The mortality rate decreased from 291.280 to 224.67 per 100,000 (EAPC = −0.57, 95% CI: −0.75 to −0.4), showing a downward trend. It is worth noting that in high-middle SDI regions, the LRI mortality rate among the elderly over 70 years old showed a slight upward trend (EAPC = 0.32, 95% CI: 0.1 to 0.54), and the change trends of other age groups were relatively flat (Figure 4. Mortality rates of LRI by age group in global and SDI regions from 1991 to 2021).

**Table 1:** The lower respiratory infection (LRI) deaths and mortality and their temporal trends from 1991 to 2021.

Characteristics	All-Age Deaths			Age-Standardized Mortality		
	Deaths (95%UI)		Percentage Change (%)	Deaths per 100,000 People (95%UI)		EAPC (95%CI)
	1991	2021		1991	2021	
Global	2,976,802 (2,710,043 to 3,242,877)	2,183,001 (1,979,915 to 2,360,084)	−26.67	60.57 (55.82 to 65.10)	28.67 (25.92 to 31.07)	−2.29 (−2.42 to −2.16)
Sex						
Male	1,557,418 (1,411,931 to 1,709,141)	1,172,230 (1,078,493 to 1,269,920)	−24.73	68.83 (63.88 to 73.66)	34.25 (31.42 to 37.05)	−2.15 (−2.28 to −2.03)
Female	1,419,384 (1,274,977 to 1,568,055)	1,010,771 (878,596 to 1,128,327)	−28.79	55.10 (49.83 to 60.35)	24.50 (21.24 to 27.10)	−2.46 (−2.60 to −2.32)
Socio-Demographic Index						
Low	765,386 (653,953 to 892,097)	502,759 (429,167 to 581,034)	−34.31	140.55 (125.05 to 155.09)	70.68 (62.56 to 78.62)	−2.16 (−2.28 to −2.04)
Low-middle	940,472 (844,161 to 1,040,618)	594,109 (525,288 to 658,730)	−36.83	80.46 (73.31 to 87.33)	43.12 (38.11 to 47.74)	−1.73 (−1.87 to −1.58)
Middle	758,857 (702,551 to 819,443)	542,885 (493,805 to 588,398)	−28.46	57.20 (52.89 to 61.25)	25.43 (22.91 to 27.61)	−2.50 (−2.62 to −2.37)
High-middle	241,321 (225,903 to 259,279)	241,824 (215,370 to 266,650)	0.21	28.51 (26.55 to 30.63)	13.79 (12.36 to 15.16)	−2.37 (−2.52 to −2.22)
High	268,624 (243,401 to 281,323)	299,446 (253,666 to 324,184)	11.47	24.91 (22.51 to 26.15)	12.05 (10.44 to 12.92)	−2.21 (−2.43 to −1.98)
GBD region						
High-income Asia Pacific	70,906 (63,973 to 74,545)	96,858 (78,185 to 107,835)	36.60	40.41 (35.91 to 42.70)	14.35 (12.01 to 15.72)	−2.85 (−3.15 to −2.55)
Central Asia	53,408 (50,072 to 56,962)	19,996 (17,590 to 22,430)	−62.56	62.99 (59.28 to 66.83)	23.34 (20.71 to 25.98)	−3.39 (−3.67 to −3.11)
East Asia	477,275 (422,536 to 532,819)	224,461 (187,516 to 269,818)	−52.97	59.00 (51.83 to 64.49)	14.52 (12.24 to 17.43)	−5.33 (−5.55 to −5.11)
South Asia	793,624 (696,348 to 881,322)	515,691 (452,545 to 584,513)	−35.02	73.84 (65.30 to 81.40)	39.19 (34.36 to 44.88)	−1.78 (−1.95 to −1.60)
Southeast Asia	236,686 (212,205 to 267,017)	200,357 (172,210 to 221,536)	−15.35	60.27 (53.94 to 69.58)	38.44 (32.69 to 42.59)	−0.96 (−1.12 to −0.81)
Australasia	2729 (2444 to 2924)	4335 (3539 to 4804)	58.85	12.83 (11.36 to 13.79)	6.70 (5.53 to 7.39)	−1.62 (−2.19 to −1.04)
Caribbean	16,575 (14,988 to 18,377)	16,708 (14,754 to 18,893)	0.80	54.59 (50.21 to 59.56)	32.76 (28.63 to 37.08)	−1.44 (−1.61 to −1.26)
Central Europe	27,673 (26,657 to 28,441)	33,047 (30,004 to 35,141)	19.42	23.69 (22.71 to 24.41)	15.52 (14.22 to 16.49)	−1.41 (−1.80 to −1.02)
Eastern Europe	23,810 (23,304 to 24,348)	29,780 (27,272 to 32,607)	25.07	11.88 (11.57 to 12.18)	10.26 (9.44 to 11.20)	−0.84 (−1.71 to 0.02)
Western Europe	112,482 (101,478 to 117,995)	123,133 (102,349 to 133,809)	9.47	19.46 (17.51 to 20.46)	10.01 (8.49 to 10.80)	−2.25 (−2.64 to −1.87)
Andean Latin America	32,340 (29,502 to 35,224)	29,552 (23,960 to 36,139)	−8.62	104.68 (95.92 to 114.02)	52.24 (42.32 to 63.77)	−2.09 (−2.28 to −1.90)
Central Latin America	58,926 (56,342 to 61,902)	51,353 (45,642 to 57,423)	−12.85	46.41 (44.33 to 48.09)	21.93 (19.47 to 24.65)	−2.15 (−2.58 to −1.72)
Southern Latin America	15,005 (14,106 to 15,610)	35,588 (31,486 to 38,256)	137.17	35.08 (32.64 to 36.60)	39.43 (35.00 to 42.34)	1.32 (0.98 to 1.67)
Tropical Latin America	52,854 (49,825 to 56,066)	79,308 (69,382 to 86,053)	50.05	50.22 (46.87 to 52.97)	32.81 (28.63 to 35.63)	−0.59 (−1.04 to −0.15)
North Africa and Middle East	171,657 (149,912 to 203,401)	92,168 (81,359 to 10,4416)	−46.31	50.89 (45.66 to 58.03)	22.70 (19.91 to 25.60)	−2.20 (−2.35 to −2.04)
High-income North America	77,068 (68,410 to 81,675)	60,868 (51,676 to 65,939)	−21.02	20.88 (18.58 to 22.10)	8.82 (7.63 to 9.50)	−2.57 (−2.85 to −2.30)
Oceania	5805 (4880 to 6968)	6710 (5484 to 8196)	15.59	93.14 (81.43 to 107.00)	56.8 (47.54 to 70.33)	−1.21 (−1.38 to −1.05)
Central Sub-Saharan Africa	80,969 (63,996 to 96,528)	61,639 (49,195 to 77,657)	−23.87	168.81 (139.19 to 206.26)	106.69 (81.78 to 138.59)	−1.53 (−1.60 to −1.47)
Eastern Sub-Saharan Africa	292,425 (247,132 to 346,235)	172,757 (150,812 to 195,778)	−40.92	166.33 (145.10 to 185.41)	81.94 (72.40 to 90.62)	−2.44 (−2.54 to −2.33)
Southern Sub-Saharan Africa	39,411 (35,763 to 43,052)	44,082 (39,086 to 49,141)	11.85	95.53 (86.15 to 106.21)	76.29 (67.95 to 84.33)	−0.22 (−0.83 to 0.4)
Western Sub-Saharan Africa	335,175 (277,410 to 393,016)	284,611 (221,050 to 354,111)	−15.09	151.17 (134.02 to 169.41)	85.46 (71.01 to 99.8)	−1.68 (−1.84 to −1.52)

### 3.3. Aetiology for LRI-Related Deaths

*Streptococcus pneumoniae* remains the leading cause of LRI-related deaths. In 2021, there were 505,268 LRI deaths due to pneumococcal infection, while the second leading cause of death was *Staphylococcus aureus*, responsible for 423,837 deaths, accounting for 23.16% and 19.43% of all LRI-related deaths that year, respectively (Table S6). Additionally, previous reports only mentioned 4 causes of LRI-related deaths, but this study adds 14 more causes. The number of LRI deaths for each aetiology is shown in Table S6. We also compiled the number of deaths caused by different aetiologies across various age groups from 1991 to 2021. From Table 2 and Table S6, it is evident that *Streptococcus pneumoniae* is the leading cause of LRI-related deaths, particularly with high death tolls in the <5 years and 70+ years age groups.

**Table 2:** The number of deaths in different age groups caused by various aetiologies from 1991 to 2021.

Aetiology	<5 Years (Deaths)	5–14 Years (Deaths)	15–49 Years (Deaths)	50–69 Years (Deaths)	70+ Years (Deaths)
*Streptococcus pneumoniae*	12,931,096	841,690	1,966,632	2,356,416	5,965,592
*Staphylococcus aureus*	2,340,802	208,621	556,887	1,381,636	5,209,719
Respiratory syncytial virus	2,930,303	15,740	47,458	62,085	202,771
*Pseudomonas aeruginosa*	1,084,913	52,671	150,715	431,599	1,443,515
Polymicrobial	487,242	10,552	23,180	61,710	150,980
Other viral aetiologies of LRI	2,782,678	104,866	296,885	333,175	1,099,548
Other bacterial pathogen	694,773	107,320	244,698	703,779	2,211,571
Mycoplasma	962,562	102,463	251,497	220,689	484,972

## 4. Discussion

In this study, we comprehensively described the changes in the deaths and mortality rates of LRIs from 1991 to 2021, as well as their distribution across different regions, age groups, and aetiological data. The results indicate a declining trend in global deaths and ASMR due to LRIs during the 1991–2021 period. However, LRIs remain one of the leading global disease burdens [16,17]. The number of deaths attributed to LRIs in 2021 was still high, particularly among populations in low SDI regions, children under 5 years old, and elderly individuals over 70 years old, who face higher risks of mortality. We suggest that countries should intensify efforts in the prevention and treatment of respiratory diseases among children and the elderly. Previous studies have shown that the COVID-19 pandemic and the associated control measures for respiratory infections have affected the epidemic patterns of many respiratory diseases [11,18,19]. This study updates our previous results on the disease burden of lower respiratory infections in 2019, extending the analysis to 2021, following the COVID-19 pandemic.

From 1991 to 2021, the ASMR for LRIs showed a declining trend. In 2021, the global LRI ASMR was 28.67 per 100,000, marking a substantial decline from 60.57 per 100,000 in 1991; this trend is similar to previous studies [12]. However, the study indicates that the ASMR in low SDI and low-middle SDI regions was significantly higher than that in middle SDI and high-middle SDI regions. Additionally, among the elderly population aged over 70 years, the mortality rate due to LRIs has declined slowly or even shown a slight upward trend. LRI remains one of the leading causes of death among individuals aged over 70 years, highlighting the need for an increased focus on this demographic. As economic and social development accelerates the trend of an aging population, according to a WHO report [20], by 2030, one-sixth of the world’s population will be aged 60 and above. From 2020 to 2030, the population aged over 60 will increase from 1 billion to 1.4 billion. By 2050, the global population aged over 60 will double to 2.1 billion, with 80% of these elderly individuals residing in low- and middle-income countries, potentially leading to a more severe burden of LRI-related deaths among the elderly. Therefore, effective measures should be implemented globally and nationally to address the disease burden associated with respiratory infections in the context of an aging population [21].

Between 1991 and 2021, the global number of deaths attributable to LRIs also showed a declining trend. This study identified that LRIs resulted in 2.49 million deaths in 2019 according to Kang et al. [12]. However, the 2021 GBD database reports a revised 2019 estimate of 2.55 million deaths. Our analysis adopts this updated GBD figure. The number of deaths subsequently declined to 2.28 million in 2020 and 2.18 million in 2021, representing a marked reduction of approximately 14.51% from 2019 to 2021. The observed downward trend may be attributable to advancements in healthcare technology, the development of and widespread vaccination against various pathogens, and the implementation of non-pharmacological interventions—such as mask wearing, stay-at-home orders, and social distancing during the COVID-19 pandemic from 2020 to 2021 [19,22] which likely reduced LRI transmission, thereby decreasing the number of deaths and mortality rates associated with LRIs [22]. Additionally, the number of deaths among children under 5 years old demonstrated a rapid decline, which may be associated with the Acute Respiratory Infection Control Programs [23] initiated by the WHO in the 1980s, the Integrated Management of Childhood Illness (IMCI) programs [24] in the mid-1990s, vaccination campaigns, improvements in nutrition, the use of antibiotics, and enhancements in maternal education, prenatal care, and interventions targeting mothers and children in several countries [25]. Despite these multifaceted efforts to reduce child deaths from LRIs, LRI remains a leading cause of mortality among children, particularly in economically underdeveloped regions [6,26]. Consequently, countries should allocate greater attention and healthcare resources to vulnerable populations, including children and the elderly.

Etiologically, *Streptococcus pneumoniae* remains the primary cause of global deaths due to LRIs, consistent with previous research findings [12,26], followed by *Staphylococcus aureus* and *Klebsiella pneumoniae*. Therefore, intensifying prevention and control efforts for *Streptococcus pneumoniae*, improving diagnostic accuracy, reducing antibiotic misuse to delay the emergence of drug-resistant bacteria, and other measures can significantly decrease deaths caused by LRI [16,26]. Currently, the administration of pneumococcal conjugate vaccines and Respiratory Syncytial Virus (RSV) vaccines can reduce some LRI-related deaths. However, due to the high cost of RSV vaccines [21], they are inaccessible in most low- and middle-income countries (LMICs) [27]. Currently, no effective vaccine exists for *Staphylococcus aureus*, thus, increasing the healthcare resources dedicated to the development of an effective *Staphylococcus aureus* vaccine could substantially reduce the disease burden caused by LRIs. Additionally, influenza accounts for a significant number of deaths across all age groups, particularly in vulnerable populations such as those under 5 years and over 70 years old, causing substantial mortality. Therefore, accelerating vaccine research and development, promoting vaccination, and ensuring universal access to influenza vaccines for vulnerable populations can effectively prevent and control deaths related to LRI [28]. By the end of 2020, 151 and 192 member states had introduced pneumococcal vaccines and *Haemophilus influenzae* type b (Hib) vaccines, with an estimated global coverage of the third dose at 49% and 70%, respectively [29]. The Global Vaccine Action Plan [30,31] aimed to achieve 90% vaccination coverage for all evaluated vaccines by 2020, yet only 11 countries and territories met this target. Therefore, regional policymakers should formulate relevant policies to accelerate vaccination programs in countries and regions with a high burden of LRI, thereby reducing deaths and the disease burden caused by LRIs.

There are some limitations in our study. First, the availability of GBD data limited the analysis of this study; we could not analyse countries which were not available in the GBD database. Second, due to the global COVID-19 pandemic in 2020–2021, deaths caused by LRIs might have been confounded with deaths from COVID-19 pneumonia. Third, the results of our study relied on the availability and quality of primary data on LRIs in GBD 2021, and like all GBD studies, the limitations of the GBD methodology might cause bias in our estimates.

In conclusion, this study indicates a declining trend in both the number of deaths and ASMR due to LRIs globally over the past 30 years. However, the number of deaths and mortality rates remain alarmingly high in low SDI regions, particularly in the Saharan Africa region, as well as in the age groups of under 5 years and over 70 years. These vulnerable populations warrant enhanced attention. Consequently, in the future prevention and control of LRIs, countries should prioritize these vulnerable groups by intensifying the research and use of effective vaccines, particularly those targeted at children and the elderly. Efforts should also be made to improve LRI prevention and control capabilities, ensure precise diagnosis and treatment, and avoid antibiotic misuse that leads to drug-resistant infections. A combination of measures is necessary to reduce the overall disease burden of LRIs.

## Data Availability

The datasets generated during and/or analyzed during the current study are available in the GBD repository, [http://ghdx.healthdata.org/gbd-results-tool] (accessed on 5 November 2024).

## Supplementary Materials

The following supporting information can be downloaded at: https://www.mdpi.com/article/10.3390/v17070892/s1, Table S1: SDI quintiles for countries estimated in GBD 2021. Table S2: Age standardized rate. Table S3: 1991-2021 LRI deaths change. Table S4: The estimated annual percentage changes among 204 countries and territories. Table S5: The number of LRI deaths among different age groups. Table S6: The number of LRI deaths for each aetiology.

## Abbreviations

The following abbreviations are used in this manuscript:

LRI	Lower Respiratory Infections
EAPC	Estimated Annual Percentage Changes
ASMR	Age-Standardized Mortality Rate
SDI	Socio-Demographic Index
